# Knowledge of Obesity and the Elements of a Healthy Diet Among Secondary School Students

**DOI:** 10.3390/children12121628

**Published:** 2025-11-30

**Authors:** Karolina Małgorzewicz, Andrzej Wasilewski, Dominika Myśliwczyk, Małgorzata Myśliwiec, Sylwia Małgorzewicz, Eliza Wasilewska

**Affiliations:** 1 Student Collegium Medicum in Bydgoszcz, Faculty of Medicine, Nicolaus Copernicus University in Toruń, 85-089 Bydgoszcz, Poland; 330765@stud.umk.pl; 2 Student Scientific Association of Medical Chemistry and Immunochemistry, Faculty of Medicine, Wroclaw Medical University, 50-345 Wrocław, Poland; andrzej.wasilewski@student.umw.edu.pl; 3 Department of Paediatrics, Diabetology and Endocrinology, Medical University of Gdańsk, 80-210 Gdansk, Poland; dominika.mysliwczyk@gumed.edu.pl (D.M.);; 4 Department of Clinical Nutrition, Medical University of Gdańsk, 80-210 Gdansk, Poland; 5 Department of Allergology, Medical University of Gdańsk, 80-210 Gdansk, Poland; ewasilewska@gumed.edu.pl

**Keywords:** adolescents, nutrition knowledge, dietary fiber, omega-3 fatty acids, obesity

## Abstract

**Highlights:**

**Adolescent obesity remains a major and growing public health concern.**
  The study emphasizes the need for early educational interventions to prevent obesity among adolescents.

**Moderate overall nutritional knowledge was observed among high school students.**
  Only two-thirds of respondents recognized obesity as a disease.

**Knowledge of specific nutrients was limited.**
  Only 27% of students correctly identified dietary sources of omega-3 fatty acids, while 60% demonstrated an accurate understanding of dietary fiber.

**Academic profile was the strongest predictor of nutrition knowledge.**

**Abstract:**

Adolescent obesity is a growing public health concern, as poor dietary patterns contribute to nutrient deficiencies. In particular, dietary fiber and omega-3 fatty acids are underconsumed yet critical for cardiometabolic and mental health. Objective: The objective of this study was to assess high school students’ knowledge of (1) obesity as a disease, (2) the role of fiber, and (3) omega-3 fatty acids, and to identify sociodemographic predictors of this knowledge. Methods: A cross-sectional survey was conducted in January–March 2024 among 205 students (aged 14–19) from public high schools in Tri-City, Poland. Knowledge was assessed using an adapted part of the KomPAN questionnaire. Logistic regression models examined associations between knowledge and demographic variables (age, gender, academic profile). Results: Overall, 66.8% of students recognized obesity as a disease, 27% correctly identified omega-3 sources, and 60% demonstrated accurate knowledge regarding dietary fiber. The academic profile was the strongest predictor. Compared with students in Natural Sciences tracks, those in other educational tracks demonstrated significantly lower probabilities of providing correct responses (fiber: OR = 0.38, 95% CI: 0.21–0.71; omega-3: OR = 0.41, 95% CI: 0.23–0.76; obesity: OR = 0.47, 95% CI: 0.25–0.90). Age and gender were not significant predictors. Conclusions: Nutrition knowledge among adolescents is moderate, with notable gaps in understanding the specific components of a healthy diet such as dietary fiber and omega-3 fatty acids. Tailored educational interventions, combined with supportive school environments, may improve knowledge and promote healthier dietary behaviors.

## 1. Introduction

Childhood and adolescent obesity are among the most pressing global public health challenges of the 21st century. According to the World Health Organization (WHO), more than 390 million children and adolescents aged 5–19 years were overweight in 2022, including over 160 million classified as obese, a significant increase from just 4% in 1990 [1]. By 2030, an estimated 9% of children aged 5–14 worldwide will be living with obesity [2].

Obesity in youth significantly increases the risk of metabolic syndrome, insulin resistance, hypertension, cardiovascular disease, and psychosocial problems. It is also associated with lower life expectancy and an increased health system burden [3,4]. In Poland, overweight and obesity affect over 20% of boys and 15% of girls in the adolescent population [4].

Adolescence (ages 14–19) is a critical life stage marked by growing autonomy and the establishment of long-term behavioral patterns, including dietary habits. As adolescents gain independence from parental oversight, they begin to make their own food choices, which often reflect a growing preference for energy-dense, nutrient-poor foods, including fast food, sweetened beverages, salty snacks, and ultra-processed products [5,6]. Social media and peer influence reinforce these habits by promoting fad diets, unhealthy food trends, and celebrity-endorsed marketing [7,8].

In the context of the highly processed, low-quality diets of teenagers, two groups of nutrients are consumed less and less frequently, even though they are crucial: dietary fiber and omega-3 fatty acids. These are primarily obtained from vegetables, fruits, cereal products, and fish, respectively. Fiber promotes gastrointestinal health, supports weight regulation, modulates glycemia and lipidemia, and contributes to satiety. Omega-3 fatty acids (e.g., EPA and DHA) are essential for brain development, cardiovascular protection, and anti-inflammatory activity [9,10].

These components were selected for analysis based on: their underrepresentation in typical adolescent diets, which often lack whole grains, vegetables, and fatty fish; their scientifically established role in preventing obesity, cardiometabolic disease, and even mental health disorders; previous reports of low awareness among adolescents despite public discussion of “superfoods” and diet trends; and their applicability to nutrition education programs, including school-based meals and workshops. There are relatively few studies assessing nutritional knowledge and its components among adolescents, despite their importance for the prevention of lifestyle-related diseases. Existing studies indicate low levels of nutritional knowledge in this age group [11]. Importantly, research specifically addressing adolescents’ understanding of obesity as a disease is lacking.

### Aim of the Study

The main objective of the study was to assess the knowledge of secondary school students about obesity and selected elements of a healthy diet.

Specific objectives were:

1. To identify students’ knowledge of the risk factors and consequences of obesity;
2. To evaluate the knowledge of dietary sources and the role of dietary fiber in diet and weight control;
3. To evaluate the knowledge of dietary sources and functions of omega-3 fatty acids;
4. To analyze differences in the level of knowledge based on gender, age, and class profile (educational track);
5. To indicate areas that require nutritional education interventions.

## 2. Materials and Method

### 2.1. Study Design

This cross-sectional study was conducted between January and March 2024 among students from public high schools in the Tri-City area of Poland (Gdańsk, Sopot, Gdynia). All these were from large cities with more than 100,000 inhabitants. The study was part of a student-led project covering schools in the Tri-City area. Data collection for this cross-sectional study involved the use of an anonymous online questionnaire. The accuracy and reliability of the questionnaire was assessed with Cronbach’s alpha coefficient (value at the level of 0.55). The study was conducted with the approval of the Bioethics Committee (No. NKBBN/559/2015). Participants provided informed consent to take part in the study online. The research took the form of an online survey, which was accessible to parents and students from eight schools located in the Tri-City area.

### 2.2. Data Collection

The link to the online survey was shared through social media (Facebook, Instagram and WhatsApp), and by personal contacts of the research group members. The respondents were recruited voluntarily. We also asked the participants to share the study link to increase the number of persons who received the invitation to the study and thus increase study participants. Before starting the survey, the participants were acquainted with a short description of the research and its purpose. They were also informed of anonymity and confidentiality. The respondents did not provide their names or contact details and were able to complete the survey at any stage of its completion. Responses were saved only after clicking the “send” button after completing the survey. Participants were not rewarded for participating in the study.

### 2.3. Participants

Participants were recruited from eight public high schools with various academic profiles: (1) Natural Sciences (biology-chemistry intensive); (2) STEM (mathematics-based profiles, including mathematics-physics and mathematics-geography); (3) Humanities/Social Sciences (humanities-focused profiles); and (4) General (non-specified or mixed profiles). The inclusion criteria for the study were attendance at a school participating in the project and consent to participate.

**Figure 1:**
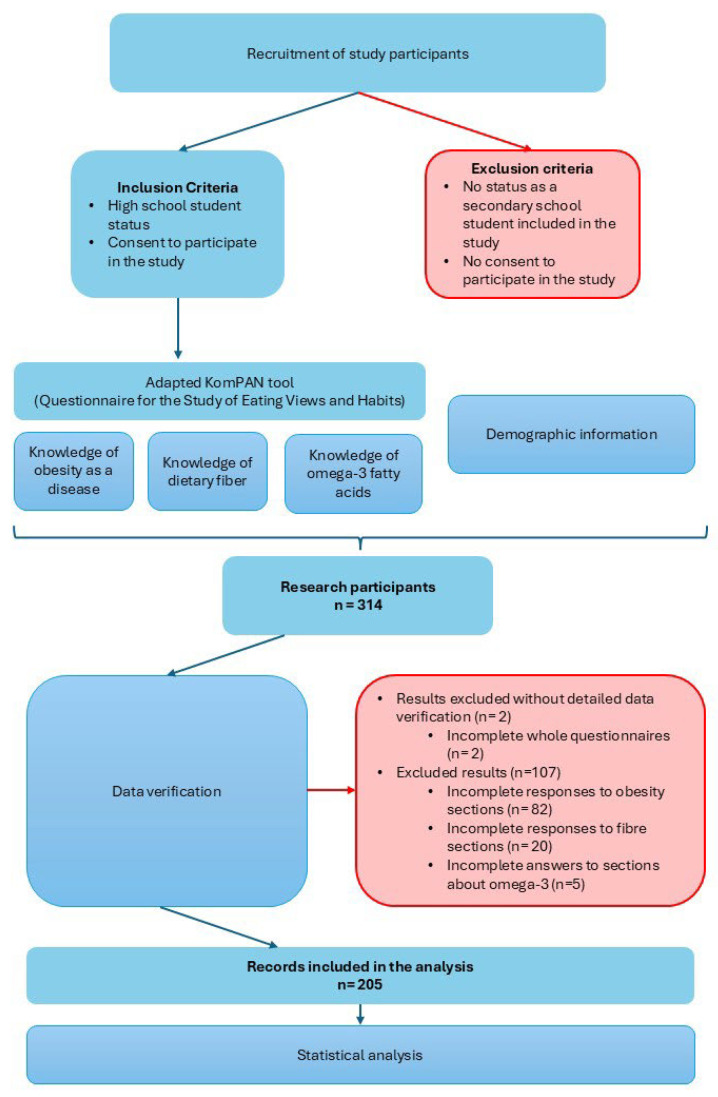
Flowchart of participant inclusion, exclusion and final sample.

Participation was voluntary and anonymous. Of the total 312 surveys, we rejected 107 due to incomplete responses. Finally, a total of 205 students aged 14–19 years participated in the study (Figure 1).

### 2.4. Research Tool

The questionnaire assessed participants’ nutritional knowledge and beliefs about healthy eating, based in part on the KomPAN tool (Questionnaire for the Study of Eating Views and Habits), developed and validated by the Polish Academy of Sciences for use in populations aged 16–65 [12,13]. The authors adapted the tool to include questions on obesity, dietary fiber, and omega-3 fatty acids. Ten questions taken from the KomPAN questionnaire referred to the section “Beliefs about food and nutrition.” In this section, students could choose one answer: true, false, or I don’t know. For some questions, multiple answers could be selected (e.g., sources of fiber, sources of omega-3 fatty acids, causes and methods of treating obesity). The final version consisted of 21 closed-ended questions divided into four thematic blocks: 1. Knowledge of obesity as a disease; 2. Knowledge of dietary fiber; 3. Knowledge of omega-3 fatty acids; and 4. Demographic information (See Supplementary file).

### 2.5. Variables

The following constructions were operationalized into measurable variables:

- Obesity awareness: recognition of obesity as a disease (yes/no);
- Knowledge of dietary fiber: correct identification of sources and functions (binary coding);
- Knowledge of omega-3 fatty acids: correct identification of sources and functions (binary coding);
- Demographics: age (continuous, years), gender (female, male, other), and school profile (Natural Sciences, STEM, Humanities/Social Sciences, General).

### 2.6. Statistical Analysis

Data were processed and analyzed using Python version 3.11 (Python Software Foundation, Beaverton, OR, USA). The following libraries were applied: pandas (v. 2.1) for data management, statsmodels (v. 0.14) for logistic regression, and matplotlib (v. 3.8) for visualization. Descriptive statistics (mean, standard deviation, frequencies, and percentages) were calculated for all variables. Logistic regression models were constructed to identify predictors like gender, age and class profile of recognition of obesity as a disease, knowledge of dietary fiber, and knowledge of omega-3 fatty acids. Results were reported as odds ratios (OR) with 95% confidence intervals (CI). A *p*-value < 0.05 was considered statistically significant.

## 3. Results

### 3.1. Study Population

The total sample initially included 314 high school students. After removing incomplete or duplicate records, 312 valid responses were retained. However, due to partial missing data in topic-specific questions, the final analytic sample for multivariable models consisted of 205 students (Figure 1).

Table 1 presents the demographic characteristics of the analyzed group. The mean age was 16.44 years (SD = 1.01), and 60.5% of participants were female. The most common academic profile was STEM (40.5%), followed by Natural Sciences (25.4%) and Humanities (24.4%).

### 3.2. Students’ Knowledge

#### 3.2.1. Obesity

Analysis of the survey responses revealed that most of the participants demonstrated a high level of awareness regarding obesity and body mass index (BMI). Specifically, 94.1% (n = 193) reported familiarity with the concept of BMI, 70.7% (n = 145) were able to calculate it, and 68.3% (n = 140) knew the BMI cut-off value for obesity. Additionally, 66.8% (n = 137) recognized obesity as a disease, while 74.1% (n = 152) could determine an appropriate body weight. Most respondents (93.0%, n = 190) agreed that obesity negatively impacts quality of life, and 93.7% (n = 192) rejected the notion that it always results from poor diet (see Table 2).

**Figure 2:**
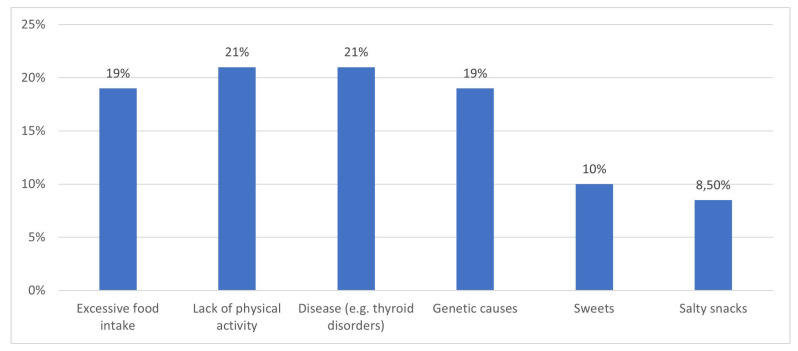
Respondents’ knowledge of the primary causes of obesity.

Figure 2 presents respondents’ perceptions of the primary causes of obesity. The most frequently reported factors were lack of physical activity and endocrine disorders (e.g., thyroid disease), each indicated by 190 participants, representing 92.7% of the study sample. Excessive food intake and genetic factors were identified by 175 respondents (85.4%). Less frequently reported causes included consumption of sweets (92 respondents, 45%) and salty snacks (82 respondents, 40%). These findings indicate that students acknowledge both lifestyle-related and biological determinants of obesity, with particular awareness of the role of medical conditions.

#### 3.2.2. Knowledge of Obesity Treatment

Students identified the primary approaches to obesity management, in order of prevalence, as: dietary modification (91%, n = 188); physical activity (90%, n = 187); bariatric surgery (54%, n = 111); psychotherapy (33%, n = 68); and pharmacotherapy (22%, n = 45). A total of 66% (n = 135) of respondents identified two or three possible options for treatment.

#### 3.2.3. Dietary Fiber

**Figure 3:**
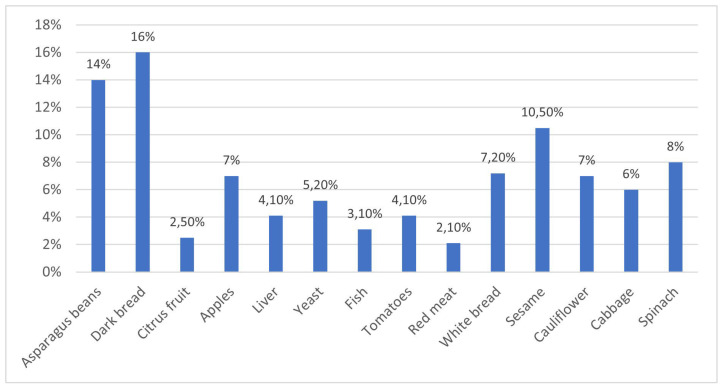
Respondents’ knowledge of dietary sources of dietary fiber.

The survey revealed notable deficiencies in students’ knowledge regarding dietary fiber, with only a proportion of respondents correctly identifying its functions, sources and recommended intake frequency. Dietary fiber, defined as the fraction of indigestible plant polysaccharides, plays a critical role in regulating gastrointestinal function, reducing blood glucose and cholesterol levels, prolonging satiety, and contributing to the prevention of overweight and obesity. While some participants were able to identify common dietary sources, such as vegetables, fruits, and whole-grain cereal products, 6% (n = 12) demonstrated insufficient knowledge in this area, wrongly indicating products, such as red meat, as sources of fiber (Figure 3). Furthermore,30% (n = 61) of respondents incorrectly indicated that it is enough to eat cereal products once a day and that fruit and vegetables do not need to be eaten with every meal.

#### 3.2.4. Omega-3 Fatty Acids

**Figure 4:**
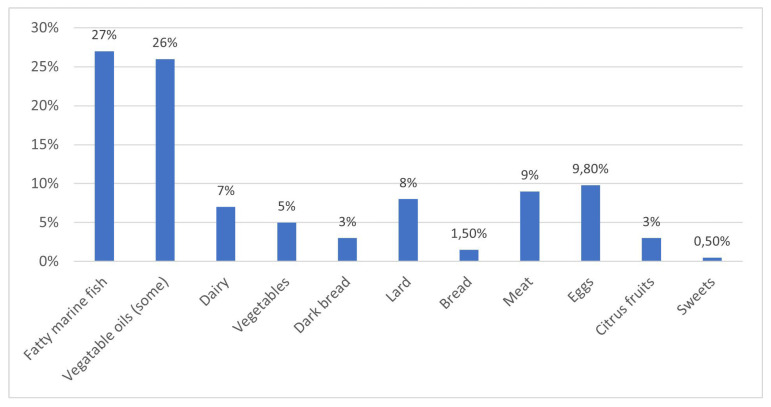
Respondents’ knowledge of dietary sources of omega-3 fatty acids.

Similarly, a considerable gap in awareness of omega-3 fatty acids was observed, with 73% (n = 169) of students unable to define them correctly. Respondents, for example, incorrectly indicated vegetables, fruits, bread, and meat as containing omega-3 fatty acids (Figure 4).

Omega-3 fatty acids are essential polyunsaturated fats found in foods such as fatty marine fish, flaxseed oil, flax seeds, eggs, and walnuts. Their regular consumption has been shown to exert anti-inflammatory effects, support brain and nervous system function, and promote metabolic health and a favorable lipid profile. The limited understanding of their role underscores the necessity of enhancing nutritional education among adolescents, particularly in the context of preventing non-communicable diseases.

In summary, the analysis showed that 66.8% of students correctly recognized obesity as a disease, 27% correctly identified omega-3 sources, and 60% correctly answered questions about dietary fiber.

Performance varied substantially by class profile. Students from Natural Sciences classes consistently scored the highest across all domains. In contrast, students from “other” profiles (e.g., STEM or Humanities tracks) demonstrated the lowest knowledge levels, particularly for dietary fiber.

There were no statistically significant differences by gender or age in the descriptive phase.

### 3.3. Predictors of Knowledge: Logistic Regression Models

Three separate logistic regression models were constructed to assess predictors of knowledge in each of the following areas: (1) fiber; (2) omega-3; and (3) obesity recognition.

Across all models, students in the “General” profile group had significantly lower odds of correct responses compared to the Natural Sciences group:

- OR = 0.38 (95% CI: 0.21–0.71) for fiber knowledge,
- OR = 0.41 (95% CI: 0.23–0.76) for omega-3,
- OR = 0.47 (95% CI: 0.25–0.90) for obesity recognition.

Other profiles (STEM, Humanities) did not show statistically significant differences. Age and gender were not significant predictors in any of the models (*p* > 0.05).

## 4. Discussion

Given the current rapid increase in excessive body weight among children and adolescents, this study assessed high school students’ knowledge of obesity and their attitudes toward food and nutrition, with a particular focus on dietary fiber and omega-3 fatty acids as key nutrients that are currently consumed in inadequate amounts.

### 4.1. Obesity

Our findings indicate that most high school students recognize obesity as a serious health and social problem. A total of 94.1% of respondents reported familiarity with the concept of body mass index (BMI), a fundamental tool for diagnosing overweight and obesity. Although 70.7% of students were able to calculate BMI, only 68.3% correctly identified the threshold for classifying obesity, suggesting gaps in health education. Regarding obesity treatment, students demonstrated awareness of a range of therapeutic approaches. The vast majority identified diet and physical activity as central components of therapy, while a considerable proportion also recognized bariatric surgery, psychotherapy, and pharmacotherapy as relevant interventions. These results suggest an encouraging level of awareness that obesity is a chronic disease requiring interdisciplinary management.

Other studies indicate that knowledge plays an important role in combating obesity among adolescents. Notably, health and nutritional literacy interventions tailored to gender, age, and region of residence are needed in school-aged children to support obesity prevention [14]. Another study demonstrated that nutrition education programs can improve knowledge, attitudes, and, consequently, dietary practices as well as BMI-for-age among adolescents [15].

### 4.2. Knowledge Regarding Components of a Healthy Diet

Our study revealed that adolescents’ knowledge of dietary fiber and omega-3 fatty acids was only moderate or low, with 60% and 27% of students, respectively, providing correct responses. These findings highlight persistent gaps in understanding the functions and sources of these nutrients, despite their well-established role in the prevention of obesity and related chronic conditions. Notably, deficiencies in knowledge were most pronounced among students in STEM tracks, suggesting that the type of educational profile strongly influences nutrition literacy.

Dietary fiber is widely recognized as a protective factor against obesity, diabetes type 2, cardiovascular disease, and colorectal cancer. It contributes to satiety, lowers postprandial glycemia, improves lipid metabolism, and supports gut microbiota diversity [2,11]. Nevertheless, our findings are consistent with previous research in Polish and European adolescents showing low awareness of fiber’s metabolic and preventive roles, and poor adherence to recommendations for whole-grain, fruit, and vegetable intake [1,16]. Knowledge gaps may be reinforced by food environments dominated by ultra-processed products, which are typically poor in fiber. Bridging this gap through education on practical food skills, such as by reading labels or identifying high-fiber alternatives, could be a cost-effective intervention for health promotion.

Similarly, omega-3 fatty acids are under-consumed and under-recognized in adolescent diets. Despite broad media coverage of “superfoods”, more than one-third of participants in our study could not correctly identify omega-3 as the essential fatty acids found in fish, seeds, and nuts. This is concerning, given that omega-3 fatty acids (particularly EPA and DHA) are critical for cardiovascular protection, neurocognitive development, and anti-inflammatory activity [10,17]. Deficient intake during adolescence may have long-term implications, not only for cardiometabolic health but also for mental well-being, as emerging studies suggest links between omega-3 status and depressive symptoms in youth [18].

In our study, students demonstrated a moderate level of nutrition knowledge—68.8% correctly identified obesity as a disease, 39% were familiar with omega 3 fatty acids, and 60% had accurate knowledge about dietary fiber. These results align with findings from the HELENA study, where the average nutrition knowledge score among adolescents from several European countries was approximately 60% [19]. The HELENA results emphasize that adolescents’ nutrition knowledge is generally insufficient and tends to be associated with socioeconomic background [19].

### 4.3. Profile of Education as a Predictor of Knowledge

The strongest predictor of knowledge in our study was the academic profile. Students from Natural Sciences (biology-chemistry) tracks scored significantly higher than their peers, while students from the other tracks had markedly lower odds of correct responses (OR ≈ 0.4). These findings are consistent with previous reports, where lower nutrition knowledge is frequently observed among students from lower educational or socioeconomic backgrounds [19].

### 4.4. Knowledge-Behavior Gap

Although nutrition knowledge was moderate, its translation into behavior remains poor. Prior research emphasizes that information alone is insufficient; motivation, beliefs, and practical food decision skills are also critical. According to the Health Belief Model, awareness must be accompanied by a perceived threat and belief in the effectiveness of change to result in behavior modification [18,20].

### 4.5. School Environment and Nutrition Education

Meta-analyses suggest that school-based nutrition interventions can significantly improve knowledge, dietary quality, and even health indicators, such as BMI and physical activity, up to 6–9 months post intervention [5].

Additionally, health education frameworks, such as UNESCO/WHO’s FRESH (Focusing Resources on Effective School Health), recommend embedding health promotion directly into the school curriculum and environment [21]. Countries, such as Japan, have demonstrated that implementing school lunch programs contributes not only to better dietary quality but also reduces inequalities in fruit and vegetable intake among children [16].

### 4.6. Critical Gaps in Dietary Knowledge

The knowledge gap concerning fiber and omega-3—present in 30–40% of our sample—is particularly concerning, as these nutrients are scarce in ultra-processed foods. Other studies show that adolescents across Europe often do not meet intake recommendations for whole grains, vegetables, and oily fish, placing them at risk of long-term cardiometabolic complications [19].

### 4.7. Limitations

This pilot study has several limitations. The sample size was relatively small. The lack of data on other potentially relevant factors, such as parental education level, household income, and food accessibility, may limit the generalizability of the findings. Furthermore, the online nature of the survey resulted in lower reliability and meant that we were required to reject incomplete responses. In addition, the study’s local scope makes it difficult to extrapolate the results to the broader population. Furthermore, the cross-sectional design precludes establishing causal relationships. Nevertheless, the study provides valuable insights by highlighting knowledge gaps among high school students in urban areas.

## 5. Conclusions and Recommendations

The study highlights the need to incorporate education about healthy eating principles and the prevention of non-communicable diseases for adolescents. Educational programs targeting students across all academic tracks are recommended. Effective nutrition education ought to encompass both the general principles of a healthy diet and specific components, such as dietary fiber and omega-3 fatty acids, together with their dietary sources. School-wide strategies-including policy-level interventions and environmental modifications (e.g., improved access to healthy foods within schools) may substantially enhance knowledge retention and foster the adoption of long-term healthy behaviors. The forthcoming introduction of comprehensive health education into the Polish school system is expected to reduce inequalities in nutritional knowledge and to strengthen the health-related competencies of secondary school students.

## Figures and Tables

**Table 1:** Demographic characteristics of study participants included in regression analysis (n = 205).

Variable	Value
Participants (n)	205
Gender Female/Male n (%)	124/81 (60.5%/39.5%)
Mean age (y.) ± SD (median)	16.44 ± 1.01 (17.0)
Profile n (%):	
STEM	83 (40.5%)
Natural Sciences	52 (25.4%)
Humanities/Social Sciences	50 (24.4%)
General	20 (9.8%)

Abbreviations: n-number, y-years, STEM (mathematics, physics, geography-based profiles); Natural Sciences (biology-chemistry intensive); Humanities/Social Sciences (humanities-focused profiles); General (non-specified or mixed profiles).

**Table 2:** Awareness and knowledge of obesity among respondents.

Question	Yes (%)	No/I Don’t Know (%)
Are you aware that obesity is a disease?	66.8	33.2
Are you familiar with the concept of BMI?	94.1	5.9
A BMI above 30 kg/m^2^ indicates obesity?	68.3	31.7
Do you know the formula for calculating BMI?	70.7	29.3
Can you determine the correct body weight?	74.1	25.9
Does obesity impair quality of life?	93.0	7.0
Is obesity always the result of an unhealthy diet?	6.3	93.7

## Data Availability

The data that support the findings of this study can be made available by the corresponding author upon reasonable request.

## Supplementary Materials

The following supporting information can be downloaded at: https://www.mdpi.com/article/10.3390/children12121628/s1, ANKIETA.

## Abbreviation

**BMI**	Body Mass Index
**WHO**	World Health Organization
**EPA**	Eicosapentaenoic Acid
**DHA**	Docosahexaenoic Acid
**STEM**	Science, Technology, Engineering, and Mathematics.
**EFSA**	European Food Safety Authority
**FRESH**	Focusing Resources on Effective School Health
**UNESCO**	United Nations Educational, Scientific and Cultural Organization
**KomPAN**	Questionnaire for the Study of Eating Views and Habits
