# Supplementary material for: Knowledge of Obesity and the Elements of a Healthy Diet Among Secondary School Students
Source: Children (Basel). 2025 Nov 30;12(12):1628. doi: 10.3390/children12121628 (PMC12731543; doi:10.3390/children12121628)
Supplement: Supplementary file 1 [file children-12-01628-s001.zip › children-3977614-supplementary.pdf]

ANKIETA

Część 1. Dane osobowe

1. Podaj swój wiek (w latach): ___________________________

2. Podaj swoją płeć (zaznacz):
   ☐ K ☐ M ☐ inne

3. Zaznacz profil klasy, do której uczęszczasz:
   ☐ biologiczno-chemiczny
   ☐ humanistyczny
   ☐ matematyczno-fizyczny
   ☐ matematyczno-geograficzny
   ☐ inny: ___________________________

Część 2. Poglądy na temat żywności i żywienia oraz wiedza o otyłości

Dla każdego stwierdzenia proszę wybrać jedną odpowiedź:
1 – prawda 2 – fałsz 3 – trudno powiedzieć

1. Produkty zbożowe wystarczy spożywać raz dziennie.
2. Owoce i/lub warzywa powinny być spożywane w każdym posiłku.
3. Ograniczenie potraw tłustych w diecie pomaga zapobiegać chorobom układu krążenia.
4. Częste spożywanie tłustych ryb morskich przyspiesza miażdżycę.
5. Pieczywo razowe zawiera większą ilość błonnika niż pieczywo jasne.
6. Owoce i warzywa są źródłem pustych kalorii.
7. Otyłość to choroba, którą należy leczyć zgodnie z wytycznymi towarzystw naukowych.
8. Czy znasz pojęcie wskaźnika masy ciała BMI?
9. Rozpoznawanie otyłości opiera się na BMI powyżej 30 kg/m^2.
10. Otyłość to zawsze skutek nieprawidłowej diety.
11. Otyłość wiąże się z pogorszeniem jakości życia.

Dla każdego następnego pytania można wybrać jedną lub więcej odpowiedzi
(chyba że zaznaczono inaczej)

---

12. Jak obliczyć BMI?

(zaznacz jedną odpowiedź)
☐ masa ciała (kg) / wzrost (m)
☐ masa ciała (kg) / wzrost^2 (m^2)
☐ masa ciała (kg) × wzrost (m)

---

13. Jakie są czynniki ryzyka otyłości?

(zaznacz jedną lub więcej odpowiedzi)
☐ brak aktywności fizycznej
☐ nadmiar jedzenia
☐ słodycze
☐ słone przekąski
☐ przyczyny genetyczne
☐ choroby (np. tarczycy)

---

14. Jakie ryzyko wiąże się z otyłością?

(zaznacz jedną lub więcej odpowiedzi)
☐ choroby serca
☐ choroby stawów
☐ choroby nerek
☐ nowotwory

---

15. Jakie są metody leczenia otyłości?

(zaznacz jedną lub więcej odpowiedzi)
☐ mniejsza ilość jedzenia
☐ dieta niskokaloryczna
☐ ruch (aktywność fizyczna)
☐ leki
☐ operacja chirurgiczna
☐ post przerywany (okresowe głodówki)

☐ głodówki

☐ psychoterapia

---

16. Pod czyją opiekę powinna udać się osoba chora na otyłość?

(zaznacz jedną lub więcej odpowiedzi)

☐ lekarza

☐ dietetyka

☐ psychologa

---

17. Które z poniższych stwierdzeń jest prawdziwe w odniesieniu do błonnika?

(zaznacz jedną lub więcej odpowiedzi)

☐ zapobiega zaparciom

☐ zmniejsza apetyt

☐ opóźnia procesy starzenia się organizmu

☐ zmniejsza stężenie cholesterolu w surowicy i poprawia tolerancję glukozy

☐ zwiększa ryzyko wystąpienia nowotworu jelita grubego

---

18. Proszę zaznaczyć produkty zawierające błonnik:

(zaznacz jedną lub więcej odpowiedzi)

☐ ciemne pieczywo

☐ białe pieczywo

☐ fasolka szparagowa

☐ jabłka

☐ kalafior

☐ kapusta

☐ owoce cytrusowe

☐ pomidory

☐ szpinak

☐ sezam

☐ drożdże

☐ mięso czerwone

☐ wątróbka

☐ ryby

19. Kwasy omega-3 to:

(zaznacz jedną odpowiedź)

☐ kwasy tłuszczowe

☐ witamina

☐ makroelement

☐ mikroelement

☐ nie wiem

---

20. Proszę zaznaczyć skutki niedoboru kwasów omega-3:

(zaznacz jedną lub więcej odpowiedzi)

☐ problemy z pamięcią

☐ zmiany zapalne stawów

☐ zwiększone ryzyko wystąpienia zawału serca

☐ zmniejsza ryzyko powstania miażdżycy

☐ zmniejsza wagę ciała

☐ wszystkie odpowiedzi są prawidłowe

---

21. Proszę zaznaczyć produkty zawierające kwas omega-3:

(zaznacz jedną lub więcej odpowiedzi)

☐ tłuste ryby morskie

☐ oleje roślinne (niektóre)

☐ jajka

☐ nabiał

☐ mięso

☐ warzywa

☐ owoce cytrusowe

☐ ciemne pieczywo

☐ pieczywo

☐ smalec

☐ słodycze
